# High-Affinity Cu(I)-Chelator with Potential Anti-Tumorigenic Action—A Proof-of-Principle Experimental Study of Human H460 Tumors in the CAM Assay

**DOI:** 10.3390/cancers14205122

**Published:** 2022-10-19

**Authors:** Dorothea M. Heuberger, Petra Wolint, Jae-Hwi Jang, Saria Itani, Wolfgang Jungraithmayr, Conny F. Waschkies, Gabriella Meier-Bürgisser, Stefano Andreoli, Katharina Spanaus, Reto A. Schuepbach, Maurizio Calcagni, Christoph J. Fahrni, Johanna Buschmann

**Affiliations:** 1 Institute of Intensive Care Medicine, University Hospital Zurich, Sternwartstrasse 14, 8091 Zurich, Switzerland; 2 Division of Plastic Surgery and Hand Surgery, University Hospital Zurich, Sternwartstrasse 14, 8091 Zurich, Switzerland; 3 Division of Thoracic Surgery, University Hospital Zurich, Sternwartstrasse 14, 8091 Zurich, Switzerland; 4 Department of Thoracic Surgery, Medical Center—University of Freiburg, Faculty of Medicine, University of Freiburg, 79106 Freiburg, Germany; 5 Division of Radiation Protection, University Hospital Zurich, Sternwartstrasse 14, 8091 Zurich, Switzerland; 6 Clinical Chemistry, University Hospital Zurich, 8001 Zurich, Switzerland; 7 School of Chemistry and Biochemistry and Petit Institute for Bioengineering and Bioscience, Georgia Institute of Technology, 901 Atlantic Drive, Atlanta, GA 30332-0400, USA

**Keywords:** copper chelation, angiogenesis, human lung cancer, CAM assay

## Abstract

**Simple Summary:**

Lung cancer is a serious burden worldwide. The growth of lung tumors depends on vessel density and intratumoral copper concentration. Copper chelating agents can reduce copper content in tumor tissue, resulting in lower vessel density and lower tumor weight. PSP-2, a very potent copper chelator, was tested on lung tumor grafts that were on-planted on the chorioallantoic membrane of the chicken embryo. We found a lower vessel density and a lower tumor weight under PSP-2 application compared to the controls. Thus, PSP-2 could be a potential therapeutic agent to treat lung cancer in the future.

**Abstract:**

Human lung cancer ranks among the most frequently treated cancers worldwide. As copper appears critical to angiogenesis and tumor growth, selective removal of copper represents a promising strategy to restrict tumor growth. To this end, we explored the activity of the novel high-affinity membrane-permeant Cu(I) chelator PSP-2 featuring a low-zeptomolar dissociation constant. Using H460 human lung cancer cells, we generated small tumors on the chorioallantoic membrane of the chicken embryo (CAM assay) and studied the effects of topical PSP-2 application on their weight and vessel density after one week. We observed a significant angiosuppression along with a marked decrease in tumor weight under PSP-2 application compared to controls. Moreover, PSP-2 exposure resulted in lower ki67^+^ cell numbers at a low dose but increased cell count under a high dose. Moreover, HIF-1α^+^ cells were significantly reduced with low-dose PSP-2 exposure compared to high-dose and control. The total copper content was considerably lower in PSP-2 treated tumors, although statistically not significant. Altogether, PSP-2 shows promising potential as an anti-cancer drug. Nevertheless, further animal experiments and application to different tumor types are mandatory to support these initial findings, paving the way toward clinical trials.

## 1. Introduction

Copper plays a critical role in activating and supporting tumorigenesis by stimulating angiogenesis [1,2]. It is involved in all steps of the angiogenic process covering initiation, endothelial cell proliferation, migration, and morphogenesis, as well as ECM remodeling and tube formation [3]. Excessive angiogenesis can arise from an imbalanced activity of pro- and anti-angiogenic cues, including increased copper levels, which may support the progression of malignant tumors. Cancer patients not only exhibit elevated copper levels in tumors [4] but generally also display higher copper serum levels [5,6,7]. Therefore, the anti-angiogenic potential of copper starvation is an important strategy in clinical oncology, where copper deprivation has been shown to compromise and reduce vascularization and thus decrease the nutrient supply of proliferating cancer cells [8]. Given these anti-angiogenic effects, copper deprivation represents a promising approach in cancer therapy.

Under physiological conditions, copper exists in the monovalent Cu^+^ (Cu(I)) or divalent Cu^2+^ (Cu(II)) oxidation states [9]. By switching between the two states, it can act as an electron transfer mediator in various enzymes, such as superoxide dismutase [10,11], cytochrome C oxidase [10], and lysyl oxidase [12]. At the same time, copper can also catalyze the formation of reactive oxygen species (ROS) by converting hydrogen peroxide to hydroxyl radicals [3,10,13]. In turn, ROS promote angiogenesis by an increase of VEGF [14] and lipid peroxidation [15]. Bioavailable copper may enhance angiogenesis through multiple avenues [16,17]. For example, copper doping induces VEGF and thus activates endothelial cells to promote angiogenesis [18], or it amplifies hypoxia effects by upregulating the expression of the hypoxia-inducible factor-1 (HIF-1) [19].

Synthetic anti-cancer agents that chelate copper with high affinity have been developed for both Cu(II) [18,20,21] and Cu(I) [22,23,24,25,26], and the application of such chelators in cancer therapy has been extensively reviewed [27,28]. For example, tetrathiomolybdate and clioquinol displayed anti-cancer activity in vitro and in preclinical animal models [29,30]. Tetrathiomolybdate was also combined with radiotherapy and proved effective in a mouse Lewis lung metastatic tumor model [31]. Recently, our research team evaluated a new high-affinity Cu(I) chelator, PSP-2 [32], for its potential anti-angiogenic activity in vitro and in ovo, i.e., in the chorioallantoic membrane assay of the chicken embryo (CAM assay) [33]. We were able to show a significant reduction in angiogenic activity when PSP-2 was applied.

Elevated copper levels appear to be a hallmark of a broad range of malignant tumors, including colorectal, breast, gastrointestinal, oral, thyroid, gall bladder, gynecologic, prostate, and lung cancers [34,35]. Moreover, a correlation between high copper serum levels and lung cancer has been reported for Asian and European populations [6]. In non-small-cell lung tumors, cancer patients exhibited approximately 50% higher copper concentrations compared to normal tissue [4]. Given the significance of lung cancer as one of the most common cancer types worldwide [36], we tested six established lung cancer cell lines for their viability upon treatment with PSP-2. Among these cell lines, H460 cells responded with the most significant reduction in viability and thus were chosen for the current study. Specifically, H460 cells were seeded in Matrigel onto the CAM assay for one week, resulting in small tumors that grew on the membrane. The tumors were then treated with PSP-2 at two different concentrations, and the tumor weight, vessel density, copper content, percentages of ki67^+^ proliferating cells and HIF-1α^+^ hypoxic cells were quantitatively assessed.

The specific hypotheses of the study were:

1. Topical PSP-2 application reduces H460 tumor weight.
2. PSP-2 treatment reduces vessel density in H460 tumors.
3. Application of PSP-2 reduces the proliferation of H460 tumor cells and enhances their HIF-1α^+^ fraction.
4. Copper amount per weight of tumor decreases in tumors treated with PSP-2.

## 2. Materials and Methods

### 2.1. Chemicals

PSP-2 (1,2-bis(bis(dimethylphosphorothioylmethyl)phosphino)ethane) was synthesized as reported previously [32]. Stock solutions of 30 mM or 60 mM in DMSO were prepared and diluted (1:1000) into the culture medium (Dulbecco’s Modified Eagle’s Medium, DMEM; Gibco, Thermo Fisher Scientific, Reinach, Switzerland). The oxidative stability of PSP-2 after storage for one year at –20 °C was verified by ^31^P-NMR. The ^31^P-NMR spectra were acquired on an Avance IIIHD spectrometer (Bruker, Ettlingen, Germany) equipped with an 11.7 T Magnet (corresponding to Larmor frequencies of 500 MHz for ^1^H and 202 MHz for ^31^P) and a Prodigy probe head (Bruker AG, Fällanden, Switzerland). Uniformly deuterated DMSO-d6 was added to the sample to shim, lock, and as a reference of the ppm scale. The ^1^H broadband decoupled ^31^P experiment with a recycle delay of 2.0 s, 256 scans, a spectral width of 294 ppm, and an acquisition time of 1.65 s was processed by zero filling to 256 k and application of 1 Hz line broadening prior to Fourier transformation and phasing.

### 2.2. Cells, Cell Culture, and Viability

The murine cell line LLC was purchased from ATCC (American Type Culture Collection; Manassas, VA, USA). The murine cell line CMT167 was a kind gift from Prof. Dr. med. Jungraithmayr. Human cell lines A549, Hop62, H2347, and H460 were obtained from Charles River (Boston, MA, USA) under a material transfer agreement with the National Cancer Institute (Bethesda, MD, USA) [37]. Passages P6–P8 were used for all experiments. All cell types were grown in DMEM, containing 10% FBS at 37 °C, 95% humidity, and 5% CO_2_. For prescreening (SI Figure S1), the effect of PSP-2 on six cell lines was determined by tetrazolium salt (3-(4,5-dimethylthiazol-2-yl)-2,5-diphenyltetrazolium bromide (MTT) assay (Sigma, Schaffhausen, Switzerland). In brief, 24 h after the cells were seeded, a culture medium with either 1:1000 DMSO (control) or 1:1000 DMSO containing 10 µM PSP-2 was added to the cells for three days. After incubation, 10 µL of the MTT reagent was added to each well. Four hours later, 100 µL of the solubilization solution was added to each well, and the plate was incubated overnight. The viability was measured with a plate reader (Infinite M Nano, Tecan, Maennedorf, Switzerland) at OD 570 nm (SI Figure S1). The value measured at OD 570 nm of the culture medium containing only 1:1000 DMSO was assumed to be 100% for each cell line (control).

For tumor experiments, approximately 10^6^ H460 cells were resuspended with 50 µL DMEM and mixed with 50 µL of Matrigel (Corning^®^ Matrigel^®^ Basement Membrane Matrix, Sigma Aldrich, St. Louis, MO, USA) CLS354230 and gently placed on the surface of the chicken embryo chorioallantoic membrane assay (CAM), which was performed as previously described [38]. Briefly, on incubation day (ID) 3.5 of the eggs, the eggshell of fertilized Lohmann white LSL chicken eggs (Animalco AG Geflügelzucht, Staufen, Switzerland) was windowed. On ID 7, H460 cells in Matrigel were placed onto the CAM within a silicon plastic ring of 1 cm in diameter. On ID 9, 11, and 13, 50 µL of a 30 µM (low-dose) or 60 µM (high-dose) PSP-2 solution in DMEM (containing 1:1000 DMSO originating from the stock solution as described in Section 2.1) was dripped onto each tumor. As a control, a solution of 50 µL DMSO, diluted 1:1000 in DMEM, was used. On ID 14, the CAM with the tumors were fixed with 4% paraformaldehyde in PBS (Kantonsapotheke, Zürich, Switzerland) overnight. All incubation steps were performed at 37 °C and 65% relative humidity. Then, the tumors were excised, deprived of surrounding tissue (from the CAM), and weighed. Afterward, the tumors were cut in half to assess histomorphometric data. For the copper content, whole tumors were immediately frozen in N_2_(l) and stored at −80 °C until further use. According to Swiss animal care guidelines (TSchV, Art. 112), no IACUC approval is required until embryonic day 14.

### 2.3. Histology

After dehydration and paraffin-embedding, the tumor specimens were cut into slices of 5 µm thickness. After removing the paraffin with xylene (Fluka, Switzerland), the sections were rehydrated (descending gradient of ethanol (Fluka, Buchs, Switzerland)) and then stained with hematoxylin/eosin (H&E) and Masson Goldner Trichrome (MGT). Micrographs of the stained sections were acquired with a Leica 6000 light microscope (Leica, Basel, Switzerland). Vessels were counted in the whole cross-section of the tumor, and the area of the corresponding cross-section was assessed with SynedraView software (*synedra View 3* version 3.1.0.3). The vessel density was calculated by dividing the number of vessels by the area of the cross-section. For low-dose PSP-2, *n* = 9 randomly selected samples were assessed; for high-dose PSP-2 and the control group, *n* = 15 and *n* = 24 were analyzed, respectively.

HIF-1α staining was performed with a mouse monoclonal antibody (Abcam, plc, Cambridge, UK; ab16066, 1:1000) using *n* = 3 randomly selected samples for low- and high-dose PSP-2, respectively, and *n* = 6 for control (with *n* = 5 fields of view (FOVs) at 100× magnification). Briefly, samples were pre-treated in PT Link (DAKO) with Envision Flex Target Retrieval Solution High pH (DAKO, K8004) and then incubated with anti-HIF-1α for 1 h. Then, the secondary antibody, consisting of labeled EnVision HRP/mouse (DAKO, K4001, dilution: RTU), was applied for 20 min. Subsequently, staining was performed in an Autostainer Link48 (DAKO), with Flex DAM and Substrate-Chromogen (DAKO, K3468) and Envision Flex Hematoxylin (DAKO, K8008). For the quantitative determination of the total cell number and HIF-1α positive cells per area, ImageJ 1.53e software was used. Briefly, after choosing the red-color channel, the threshold was set to 68 and 170 (under default B&W). Then, particles were analyzed based on size (5 to infinity) and circularity (0 to 1) to get the total number of cells. For counting HIF-1α-positive cells, the blue-color channel was selected, and the threshold was set to 0 and 144. All other parameters were set as described for determining the total cell count.

For ki67 staining, samples were pre-treated in PT Link (DAKO) with Envision Flex Target Retrieval Solution Low pH (DAKO, K8005) and incubated with monoclonal mouse anti-human ki67 MIB-1 antibody (DAKO, IR626, dilution: RTU). Then, the secondary antibody, consisting of labeled Polymer–HRP anti-mouse (DAKO, K4007, dilution: RTU), was applied. Afterward, staining was performed in an Autostainer Link48 (DAKO), with Flex DAM and Substrate-Chromogen (DAKO, K3468) and Envision Flex Hematoxylin (DAKO, K8008). For the quantitative determination of ki67 positive cells per area, all brown-stained cells were counted in the whole FOVs of the respective tumor graft and divided by the area. In ImageJ, the blue-color channel was used, and the brightness was adjusted to the maximum. The threshold was set to 0 and 75. All other parameters were set as described above for counting the total cell number.

### 2.4. MRI of the Living Chicken Embryo and CAM

To assess the volumes of the tumors, MRI was performed on the living chicken embryo with the tumor on the CAM on ID 14, seven days after grafting. MRI was performed with a 4.7 T Bruker PharmaScan instrument (Bruker BioSpin, Ettlingen, Germany) equipped with an actively decoupled two-coil system consisting of a 72 mm quadrature resonator for excitation and a 20 mm single loop surface coil for reception. The living eggs were cooled to 4 °C for 75 min in the refrigerator as a sedation measure and then gently placed into a custom-built sliding bed (for moving into the MRI instrument) and wrapped in plastic tubing for insulation to retain a constant temperature of approx. 4 °C throughout the duration of the experiment. To prevent excessive loss of moisture, the eggshell window was kept covered with a sterile plastic plate onto which the 20 mm single loop surface coil used for MRI signal detection was attached for optimal signal sensitivity.

Tumor volumes were assessed from MRI images acquired with a 3D T2-weighted TurboRARE sequence (TR 1200 ms, RARE factor 16, TE 47.5 ms, acquisition time 10 min) at a spatial resolution of 200 × 200 × 200 μm^3^. Tumor grafts were outlined manually in the MR images on orthogonal views of the 3D MRI data using custom-built software based on Matlab (Mathworks, Natick, MA, USA; Version 1.0 rev2019.

### 2.5. Copper Content in Whole Tumors

The copper content was determined by atomic absorption spectroscopy (AAS) using an AAnalyst 200 Atomic Absorption Spectrometer (Perkin Elmer, Waltham, MA, USA). Freshly harvested tumors were frozen in N_2_ (l) and stored at −80 °C for 4 months. After thawing, samples with high-dose PSP-2 and samples without PSP-2 (both *n* = 7) were used for quantitative copper analysis. One mL of tetramethylammonium hydroxide solution (TMHA, Sigma Aldrich) was added to each sample followed by heating at 60–70 °C for 1 h or until the material was completely dissolved. Internal controls were included in each run to ensure the reliability of the test results. All samples, standards, and controls were run in duplicate. The copper content was calculated by the amount of copper (μg) per g of tumor tissue. Two copper concentrations were computed, (i) based on fresh weight directly after extraction and freezing in N_2_ (l) and (ii) based on dry weight after 4 months of storage at −80 °C.

### 2.6. Statistics

Data were analyzed with StatView 5.0.1 and SPSS Statistics Version 25 (IBM SPSS, Armonk, NY, USA). First, data were analyzed for normal distribution with Kolmogorov–Smirnov (KS) and Shapiro–Wilk (SW) tests. Levene’s test was performed to confirm variance homogeneity. Providing the data were normally distributed (*p* > 0.05 in KS and SW) and the variance homogeneity was given (*p* > 0.05 Levene), parametric one-way analysis of variance (one-way ANOVA) was conducted for three groups. Pairwise comparison probabilities (*p* values) were calculated using the Fisher’s PLSD post hoc test. *p* values < 0.05 were considered as significant. Figures were plotted in GraphPad Prism 8.0 software (GraphPad Software, San Diego, CA, USA). Values were expressed as means ± standard deviations.

## 3. Results

### 3.1. Oxidative Stability of PSP-2

**Figure 1:**
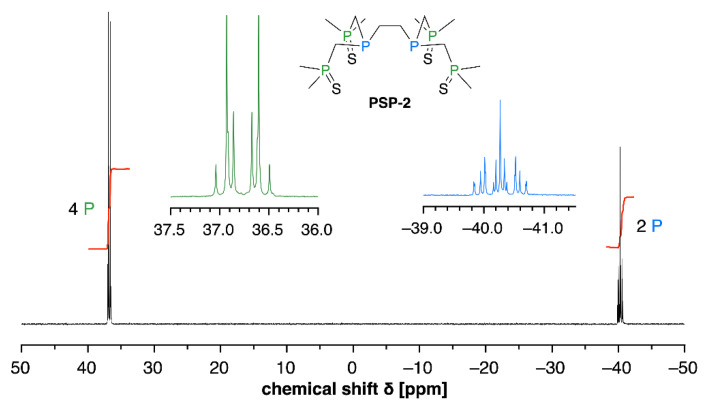
^31^P-NMR spectrum of PSP-2 to verify its integrity towards oxidation after prolonged storage in DMSO. The two signals can be assigned to the phosphine sulfides moiety centered around 37 ppm (green) and the tertiary phosphines around −40 ppm (blue), respectively [32]. The absence of additional signals confirms the oxidative stability of PSP-2 in DMSO.

The chelator PSP-2 contains two phosphorous(III) centers that might be subject to oxidation upon prolonged storage in DMSO. To verify the integrity of PSP-2, a ^31^P-NMR spectrum was acquired. As illustrated with Figure 1, the ^31^P-NMR spectrum did not show any signs of oxidation, even after storage over 1 year at −20 °C in DMSO.

### 3.2. Weight and Volume of H460 Tumors Grown on the CAM

**Figure 2:**
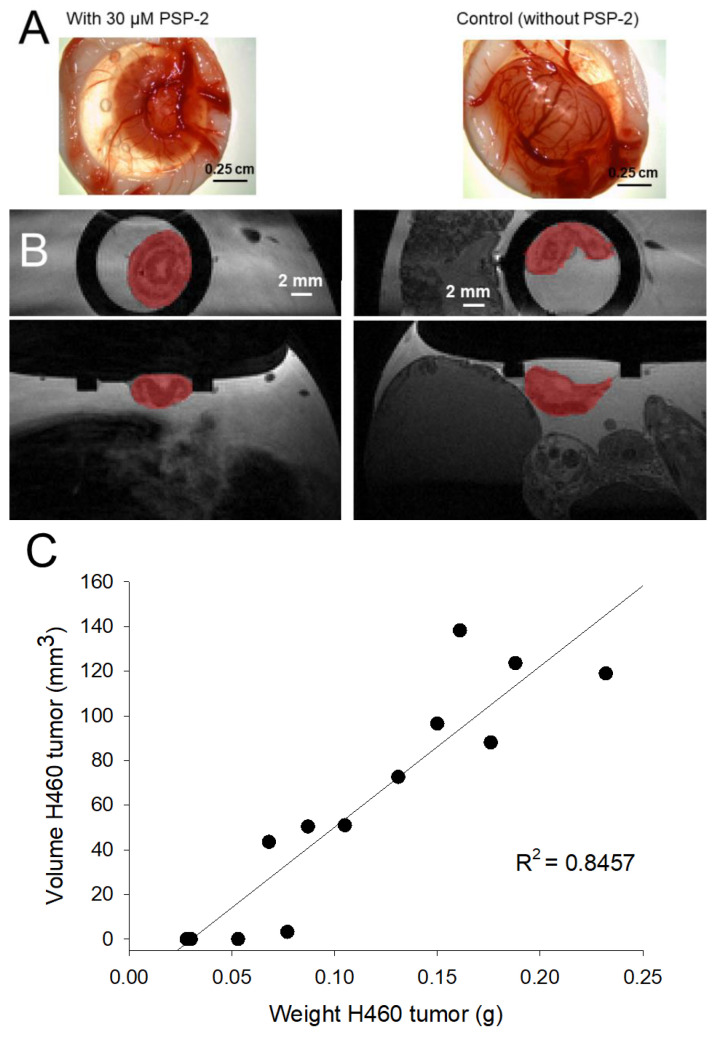
Representative images of H460 tumors after fixation (**A**) and the corresponding MRI images of H460 tumors (grown on living chicken embryos) used to assess their volumes (red colored area) (**B**). Linear correlation between the MRI tumor volume and the weight after formaldehyde fixation, excision, and deprivation of the surrounding tissue (**C**).

As a first step towards characterizing the H460 tumors grown on the CAM assay, we correlated their final weight after fixation, excision, and deprivation from the CAM tissue, with the volume determined by MRI in the living chicken embryo before fixation (Figure 2). The data established a positive correlation between tumor weight and volume (R^2^~0.85).

To assess the effect of PSP-2 on tumor growth, we compared the final weight of the tumors after fixation, resection, and deprivation of the residual CAM tissue (SI Figure S2). These data were acquired in three consecutive series for the low-dose PSP-2 treatment, with a total of *n* = 18 samples receiving PSP-2 and one series for the high-dose PSP-2 treatment with *n* = 15 samples. For the control without PSP-2, a total of *n* = 25 samples were analyzed. Because the incubation time for each of the three groups of the low-dose PSP-2 study differed by 4–8 h over a total incubation time of 84 h (3.5 days), a mean shift correction was applied prior to statistical analysis.

Kolmogorov–Smirnow and Shapiro–Wilk tests yielded *p* > 0.05 for all three groups, indicating a normal distribution of the data. Levene’s test also yielded *p* > 0.05 for all three groups, indicating variance homogeneity of the data. Hence, a one-way ANOVA with Fisher’s PLSD post hoc test was performed for pairwise comparison of the three groups. Based on this analysis, H460 tumors treated with PSP-2 had a significantly lower weight compared to controls, with *p* = 0.0061 for the low-dose PSP-2/control comparison and *p* = 0.0132 for the high-dose/control comparison. However, the low- and high-dose PSP-2 treated tumors did not differ in their average weights (*p* = 0.8965).

### 3.3. Vessel Density and Immunohistochemistry of H460 Tumors with and without PSP-2

**Figure 3:**
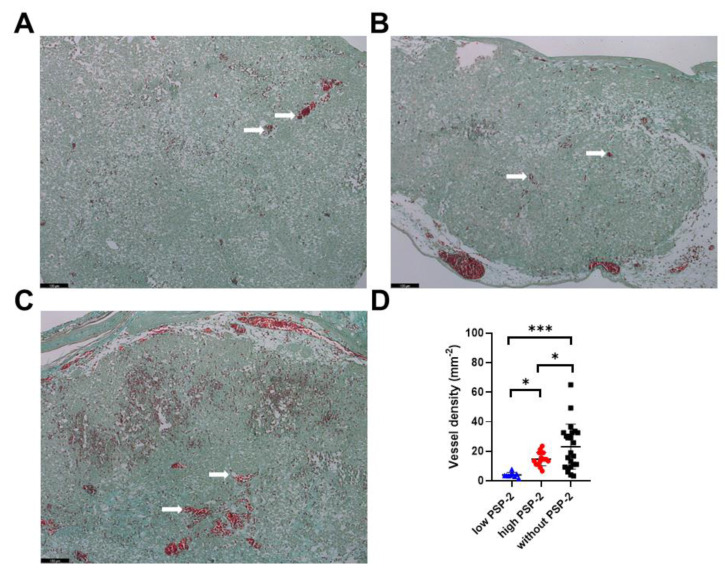
Vessel density of H460 tumors grown for 1 week on the CAM assay. Histological sections stained with Masson Goldner Trichrome for groups with low-dose PSP-2 (**A**) and high-dose PSP-2 (**B**) and without PSP-2 (**C**). Scale bars are 100 µm. White arrows indicate vessels. Vessel density (**D**) shown with mean and standard deviation. One-way ANOVA was computed and pairwise comparison probabilities *p* were calculated using the Fisher’s PLSD post hoc test, with *p* < 0.05 as significance level. Key: *p* < 0.05 (*) and *p* < 0.001 (***).

To assess the effect of PSP-2 on the vessel density, we counted the number of vessels within a given cross-section and normalized them to the cross-section area (Figure 3). There was a considerable variance in the control group without PSP-2 compared with the low- and high-dose PSP-2 groups, respectively. Nevertheless, the three groups exhibited significantly different vessel densities, with lower densities of low-dose PSP-2 compared to control and of high-dose PSP-2 compared to control. Moreover, low-dose PSP-2 tumors exhibited a lower vessel density compared to high-dose PSP-2 specimens.

**Figure 4:**
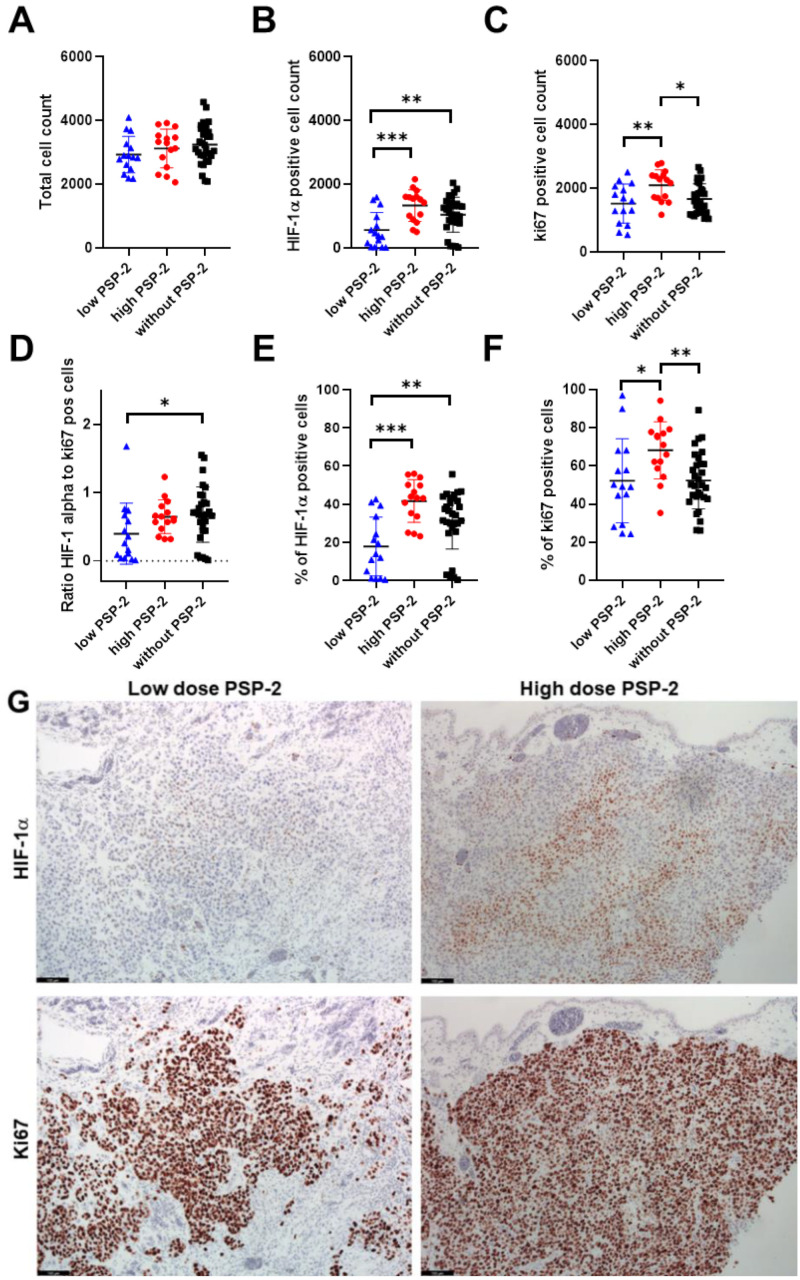
Total cell count (**A**), HIF-1α^+^ cell count (**B**), ki67^+^ cell count (**C**), ratio of HIF-1α^+^ cells to ki67^+^ cells (**D**), percentage of HIF-1α^+^ cells from total cell count (**E**), percentage of ki67^+^ cells from total cell count (**F**), and typical immunohistochemically stained sections for HIF-1α (**upper row**) and ki67 (**lower row**); low-dose PSP-2 (**left column**), high-dose PSP-2 (**right column**) (**G**). One-way ANOVA was computed and pairwise comparison probabilities *p* were calculated using the Fisher’s PLSD post hoc test, with *p* < 0.05 as significance level. Key: *p* < 0.05 (*), *p* < 0.01 (**), and *p* < 0.001 (***). Scale bar 100 µm.

In addition to the vessel density, we assessed the total cell count in five fields of view (FOVs) per sample within an area of 0.322 mm^2^. As shown in Figure 4, there was no significant difference between the three groups (Figure 4A). An analysis for HIF-1α^+^ hypoxic cells revealed that low-dose PSP-2 treated tumors exhibited a significantly lower cell count compared to high-dose PSP-2 and controls (Figure 4B). We next assessed the proliferation marker ki67 (Figure 4C). While the low-dose PSP-2 groups had a lower ki67^+^ cell count compared with the high-dose PSP-2 group, it did not show a significant difference compared to the control. In contrast, the high-dose PSP-2 groups exhibited a significantly higher ki67+ cell count compared to the control. A calculation of the HIF-1α^+^ and ki67^+^ fractions revealed the same results as the absolute cell counts (Figure 4E,F). Finally, we determined the ratio of HIF-1α^+^-to-ki67^+^ cells (Figure 4D). Only the low-dose PSP-2 groups showed a significant difference, with lower ratios compared to the control.

### 3.4. Copper Content of H460 Tumors with and without PSP-2

**Figure 5:**
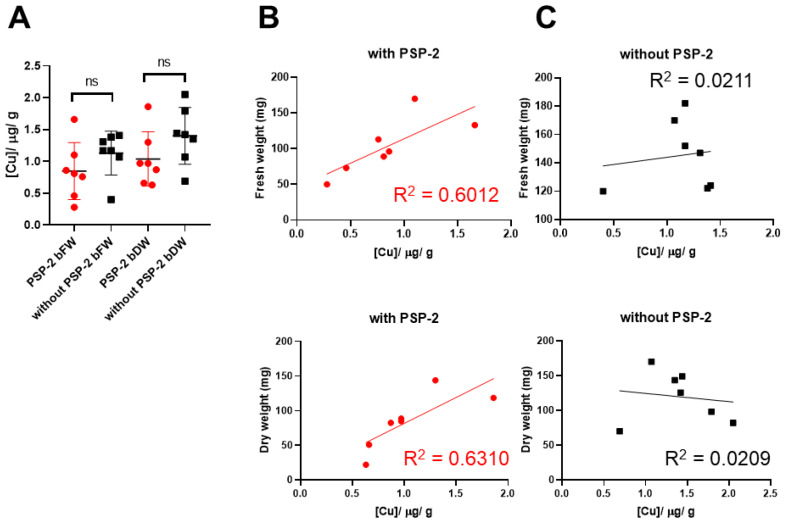
Copper content of H460 tumor samples ± high-dose PSP-2 based on the fresh or dry weight (**A**). Linear correlations between total copper content and fresh weight (**top**) or dry weight (**bottom**) of individual tumor samples treated either with PSP-2 (**B**) or without PSP-2 (**C**). Key: ns = not significant; bFW = based on fresh weight; bDW = based on dry weight; R^2^ = correlation coefficient.

To evaluate the effect of PSP-2 on copper tissue levels, we assessed the copper content in H460 tumors after digestion utilizing AAS. The total copper amount was normalized to the tumor weight, which was either determined as “fresh weight” immediately after extraction or “dry weight” after storage for 4 months at −80 °C. Because not all samples lost the same water fraction during storage, we included both weight measurements in the evaluation. As shown in Figure 5A, the average copper concentration of the high-dose PSP-2 treated tumor tissue was lower than the control group without PSP-2; however, the difference was not statistically significant. Nevertheless, a plot of the tumor weight against the total copper content of individual tumors revealed a moderately positive correlation for samples treated with PSP-2 (Figure 5B). In contrast, no correlation was observed in the control group (Figure 5C), irrespective of the weight evaluation method.

## 4. Discussion

Among micronutrients and trace elements, copper plays an essential role in tumorigenesis [1,23] and has emerged as a critical factor in angiogenesis [3,8,39]. Thus, using copper chelators that minimize the cellular copper availability represents a promising strategy in cancer therapy to attenuate, or even block, the aggressive angiogenesis found in tumors [40,41]. While a range of copper chelating agents have been described [11,22,42], their selectivity is often insufficient, as they can chelate other essential d-block metal ions, such as Fe^2+^ or Zn^2+^. In addition, their affinity might not be adequate to sequester Cu(I) within the complex biological environment of mammalian cells, where high-affinity storage proteins, such as metallothionein, maintain extremely low buffered copper concentrations [43,44].

Previously, our research group reported on the anti-angiogenic effects of the novel membrane-permeant high-affinity Cu(I) chelator PSP-2 [33], which binds Cu(I) with low zeptomolar affinity. We demonstrated that PSP-2 adversely affects tube formation of tumorigenic endothelial cells EAhy926 in vitro, impaired normal sprouting in the aortic ring assay, and resulted in suppressed branching, in favor of long, parallel arranged vessels in the chorioallantoic membrane of the chicken embryo (CAM assay) in ovo [33]. In the current study, we directly explored the potential anti-tumorigenic action of PSP-2. After pre-screening six established lung cancer cell lines (SI Figure S1), we identified the human lung tumor cell line H460 as the model of choice and grew these cells in Matrigel on the CAM surface to form small tumors with a diameter ranging between 3 and 5 mm. The tumors were either treated with two concentrations of PSP-2 or DMSO as control.

First, the oxidative stability of PSP-2 in DMSO was verified by ^31^P-NMR, confirming the integrity of phosphorous (+III), which is a prerequisite for Cu(I) chelation (Figure 1). Second, we assessed the tumor volume within the living chicken embryo by non-invasive MRI based on previously established protocols [45], and correlated these volumes with the corresponding weights of each sample (Figure 2). The positive correlation, with R^2^ ~ 0.85, allowed us to continue the study by only assessing the tumor weight at the endpoint of seven days. At that point, the tumors were excised after overnight fixation in paraformaldehyde and deprived of any surrounding tissue originating from the CAM membrane.

The main findings of this study point towards a significant decrease in tumor vessel density under PSP-2 treatment (Figure 3), accompanied by a decrease in tumor weight (SI Figure S2). Whether the reduced tumor weight was a result of the decreased H460 cell viability (SI Figure S1) or caused by lower vessel density (Figure 3), or a combination of both, remains unclear. Angiosuppression has been reported to parallel a decrease in tumor weight, as demonstrated in a rat gliosarcoma model upon copper chelation [46]. Moreover, the tumor volumes revealed a positive correlation with the microvessel density in a nasopharyngeal carcinoma mouse model [47]. Altogether, the observed decrease in the H460 tumor weight under low- and high-dose PSP-2 treatment, with concurrent significant angiosuppression, points towards an anti-cancer potential of PSP-2 via an anti-angiogenesis mechanism. A limitation of the CAM assay, however, is the relatively short time window for tumors to grow (due to restrictions by the Swiss animal care guidelines (TSchV, Art. 112)).

Along with the observed lower vessel densities in PSP-2 treated tumors (Figure 3), we also expected an increased fraction of HIF-1α^+^ cells (Figure 4), as fewer vessels may provoke a hypoxic state, at least within the core of the tissue. However, the findings proved to be more complex. Although the high-dose PSP-2 and control groups were not significantly different, the low-dose PSP-2 group revealed a significantly lower fraction of HIF-1α^+^ cells compared to both other groups, even though the total cell counts were similar for all three groups. While the fraction of HIF-1α^+^ cells was 18 ± 15% under low-dose conditions, it was 42 ± 11% under high-dose and 31 ± 15% in the control, respectively. According to the categories set up for HIF-1α^+^ cell nuclei reported by Rzepakowska et al. [48], the low-dose PSP-2 results lie in category 1, defined as 1–25%, while the high-dose and control lie in category 2, ranging between 25–50%, respectively.

Upregulation of HIF-1α has been reported to increase VEGF in bone marrow-derived stromal cells [19], among other cell types [49], which, in turn, stimulates angiogenesis. In that sense, the significantly higher HIF-1α^+^ cell fraction found under high-dose compared to low-dose PSP-2 conditions (Figure 4) could therefore account for the significantly higher vessel density found for the high-dose compared to the low-dose PSP-2 treatment (Figure 3). In accordance, Triner et al. have attributed enhanced angiogenesis in more hypoxic tumors to higher immune cell recruitment that increases pro-angiogenic ROS, among others [50]. Nevertheless, it has to be taken into account that anti-angiogenic therapy may lead to a selection of hypoxia-resistant tumor cells as a possible mechanism of therapy resistance [51]. This might, at least partially, explain why low-dose PSP-2 treated tumor cells exhibited a significantly lower hypoxia fraction (Figure 4).

Since increased HIF-1α levels stimulate enzymes responsible for cancer survival [52], it is interesting to note that despite higher vessel density and a higher percentage of HIF-1α^+^ cells, high-dose PSP-2 treated tumors experienced a similar weight reduction as low-dose PSP-2 specimens when compared to control (SI Figure S2).

An additional critical aspect is the proliferation of tumor cells. In this regard, the ki67^+^ cell fraction was higher for high-dose PSP-2 compared to control and low-dose PSP-2, but similar for low-dose PSP-2 and control (Figure 4). Relating the results of ki67 fractions to the fractions of hypoxic cells, proliferation within more hypoxic tumors (here under high-dose PSP-2) seems to be upregulated compared to the control and low-dose PSP-2. It has been reported that upregulation of HIF-1α and overexpressed VEGF are associated with higher cellular proliferation [53,54], specifically for several non-small-cell lung cancers [55]. These reports are in line with our findings. A further explanation supporting the higher proliferation found for high-dose PSP-2 treated tumors compared with the control could be based on a selection of cells with reduced vascular dependence [51], as the vessel density was lower in high-dose PSP-2 compared to the control.

Shahrzad et al. have emphasized the multifaceted relationship between tumor progression and angiogenic status [51]. Despite angiogenic blockade and hypoxia, tumors may nevertheless keep on growing, because, on the one hand, vascular dependence of cancer cells may be different, and on the other hand, cancer cell selection during progression may lead to a predominance of cells with oncogene activation. Specifically, the authors have reported on the induction of *K-ras* mutation under hypoxia [51]. In turn, *K-ras* mutation stimulates cell proliferation. Hence, our findings for high-dose PSP-2 treatment go along with these considerations, namely the increased ki67^+^ cell fraction (Figure 4) and the lower vessel density compared to the control (Figure 3).

The quantification of total copper revealed a trend towards lower concentrations in high-dose PSP-2 tumors compared to controls (Figure 5). This was expected, as copper chelation by PSP-2 should lower the copper content in tumors, providing the PSP-2 chelated copper is removed from the tumor tissue through blood circulation. Copper serum levels in tumor patients [56] and copper content in tumor tissue have been reported to be significantly higher compared to healthy tissue [57]. Here, we determined 0.85 ± 0.45 μg Cu/g tumor tissue (fresh weight) after PSP-2 application, as opposed to 1.13 ± 0.34 μg Cu/g tumor tissue without PSP-2. For the dry weight-based copper contents, the corresponding values were 1.04 ± 0.43 μg Cu/g and 1.40 ± 0.45 μg Cu/g tumor tissue, respectively. These values are very similar to the values determined by Cheng et al. for non-small-cell lung cancer tissues, with 1.518 μg Cu/g tumor tissue, compared to normal tissue with 1.012 μg Cu/g tissue [4].

Interestingly, a positive correlation was observed when PSP-2 treated tumor weight was plotted against the copper concentration (Figure 5B), while there was no such correlation in controls (no PSP-2, Figure 5C). It might be speculated that angiosuppression induced by PSP-2 restricts tumor growth to a certain extent, which might depend on the actual copper concentration within the microenvironment. Thus, smaller tumors contain less copper per gram of tumor, because less copper is associated with lower angiogenic activity. In contrast, if no copper chelating agent is present (control, no PSP-2), the tumor weight did not correlate with the copper content, and natural copper fluctuation within the tumor tissue occurs. Similar results have been reported for gliosarcoma in a rat model [46], where treatment with the copper chelator D-penicillamine [58] yielded not only a reduction in tumor weight but also a significantly decreased copper concentration [46].

## 5. Conclusions

The findings obtained when applying PSP-2 with H460 tumor grafts on the CAM point towards an anti-cancer effect of this high-affinity copper(I) chelator. Besides a reduction in tumor weight, angiosuppression previously observed in vitro was confirmed in this study in ovo. Before clinical trials can be considered, however, PSP-2 should be tested with other types of tumors and in preclinical animal models that allow for an observation period longer than 1-week, which was imposed by the CAM assay used in this study.

## Data Availability

The data can be shared upon request.

## Supplementary Materials

The following supporting information can be downloaded at: https://www.mdpi.com/article/10.3390/cancers14205122/s1, Figure S1: MTT viability assay for six lung cancer cells lines, expressed as % OD values (optical density) upon treatment with 10 µM PSP-2 in culture medium relative to untreated control cells (without PSP-2). Figure S2: Weight of H460 tumors grown for 1 week on the CAM assay.
