# Supplementary material for: High-Affinity Cu(I)-Chelator with Potential Anti-Tumorigenic Action—A Proof-of-Principle Experimental Study of Human H460 Tumors in the CAM Assay
Source: Cancers (Basel). 2022 Oct 19;14(20):5122. doi: 10.3390/cancers14205122 (PMC9600560; doi:10.3390/cancers14205122)
Supplement: Supplementary file 1 [file cancers-14-05122-s001.zip › cancers-1943194-supplementary.pdf]

Supplementary Information

High Affinity Cu(I)-Chelator with Potential Anti-Tumorigenic Action – a Proof-of-Principle Experimental Study of Human H460 Tumors in the CAM Assay

Dorothea M. Heuberger [1], Petra Wolint [2], Jae Hwi Jang [3], Saria Itani [3], Wolfgang Jungraithmayr [3,4], Conny F. Waschkies [5], Gabriella Meier-Bürgisser [2], Stefano Andreoli [2], Katharina Spanaus [6], Reto A. Schuepbach [1], Maurizio Calcagni [2], Christoph J. Fahrni [7] and Johanna Buschmann [2,*]

1. Institute of Intensive Care Medicine, University Hospital Zurich, Sternwartstrasse 14, 8091 Zurich, Switzerland

2. Division of Plastic Surgery and Hand Surgery, University Hospital Zurich, Sternwartstrasse 14, 8091 Zurich, Switzerland

3. Division of Thoracic Surgery, University Hospital Zurich, Sternwartstrasse 14, 8091 Zurich, Switzerland

4. Department of Thoracic Surgery, University Hospital Freiburg, Freiburg, Germany

5. Division of Radiation Protection, University Hospital Zurich, Sternwartstrasse 14, 8091 Zurich, Switzerland

6. Clinical Chemistry, University Hospital Zurich, Switzerland

7. School of Chemistry and Biochemistry and Petit Institute for Bioengineering and Bioscience, Georgia Institute of Technology, 901 Atlantic Drive, Atlanta, GA, 30332-0400, USA

* Correspondence: Johanna.Buschmann@usz.ch; +41442559895

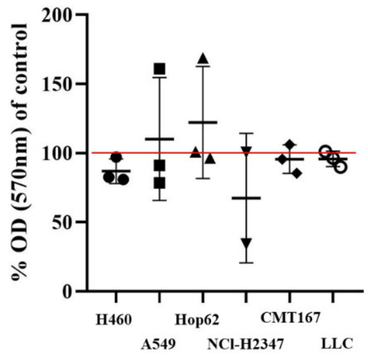

SI Figure 1: MTT viability assay for six lung cancer cells lines, expressed as % OD values (optical density), upon treatment with 10 μM PSP-2 in culture medium relative to untreated control cells (without PSP-2).

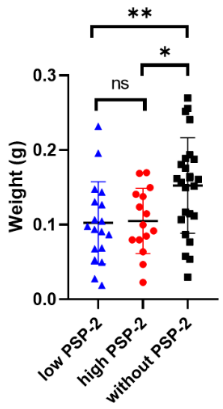

SI Figure 2: Weight of H460 tumors grown for 1 week on the CAM assay. Groups represent high-dose PSP-2 (n = 15), low-dose PSP-2 (n = 18), and control treatment without PSP-2 (n = 25); mean and standard deviation are shown. The PSP-2 treated samples had a significantly lower weight compared to the controls. One-way ANOVA was computed, and pairwise comparison probabilities p were calculated using the Fisher's PLSD post hoc test, with $p < 0.05$ as significance level. Key: $p < 0.05$ (*), $p < 0.01$ (**) and ns = not significant.
